# Medicinal Plants and Zinc: Impact on COVID-19 Pandemic

**DOI:** 10.1155/2021/9632034

**Published:** 2021-09-23

**Authors:** Zineb Jalal, Meryem Bakour, Badiaa Lyoussi

**Affiliations:** Laboratory of Natural Substances, Pharmacology, Environment, Modeling, Health and Quality of Life (SNAMOPEQ). Faculty of Sciences Dhar El Mahraz, University Sidi Mohamed Ben Abdellah, Fez, Morocco

## Abstract

The world is currently grappling with the coronavirus disease (COVID-19) pandemic, caused by severe acute respiratory syndrome coronavirus 2 (SARS-CoV-2). The infection can cause fever, a dry cough, fatigue, severe pneumonia, respiratory distress syndrome, and in some cases death. There is currently no effective antiviral SARS-CoV-2 drug. To reduce the number of infections and deaths, it is critical to focus on strengthening immunity. This review aims to conduct a comprehensive search on the previous studies using Google Scholar, ScienceDirect, Medline, PubMed, and Scopus for the collection of research papers based on the role of zinc in the immune system, the antiviral activity of zinc, the effect of zinc supplementation in respiratory infections, the therapeutic approaches against viral infections based on medicinal plants, and the role of plants' bioactive molecules in fighting viral infections. In conclusion, we highlighted the pivotal role of zinc in antiviral immunity and we suggested the bioactive molecules derived from medicinal plants as a search matrix for the development of anti-SARS-CoV-2 drugs.

## 1. Introduction

Pandemic diseases are a global concern in the current era, as they cause enormous morbidity [1]. The rise of the density of inhabitants and the fast universal urbanization lead to many challenges to global health, such as the rapid spread of infectious diseases due to the close contact between people in urban areas and the emergence of slum settlements known by the poor access to clean water and sanitation [2]. With the advent of the twenty-first century, our planet has observed the incidence of catastrophic viral epidemics, namely, severe acute respiratory syndrome (SARS-CoV) and Middle East respiratory syndrome (MERS-CoV) in the human population [3]. Currently, coronavirus disease 2019 (COVID-19) is the third most important disease of animal origin, which prevails in all corners of the world. Nearly 219 countries on all continents have been affected in less than three months by this virus [4]. After studying its clinical features, experts founded that it is quite similar to pneumonia and hence named novel coronavirus. However, in the second week of March 2020, COVID-19 has been declared as a pandemic by the World Health Organization (WHO) [5]. The causative agent of COVID-19 is severe acute respiratory syndrome coronavirus 2 (SARS-CoV-2) according to the official name of the International Committee on Taxonomy of Viruses (ICTV) [6]. To prevent the spread of COVID-19, the WHO has put in place recommendations such as frequent handwashing with soap and water or the use of a hydroalcoholic solution, stay away from anyone who coughs or sneezes, wear a mask when physical distancing is not possible, and avoid touching the eyes, nose, or mouth. In case of coughing or sneezing, cover the nose and mouth with the bend of the elbow or tissue [7]. The most common symptoms of COVID-19 are fever, cough, loss of smell, and myalgia [8]. Currently, existing antiviral drugs such as lopinavir, chloroquine, nitazoxanide, ritonavir, hydroxychloroquine, tocilizumab, and azithromycin have been used, which tend to reduce replication and viral load [9]. Furthermore, around the world, among hundreds of vaccines that have been tested in clinical trials, some of them have already been approved and the vaccination in several countries has already started [10].

In addition to that, zinc occupies an important place in the therapeutic strategy of the disease [11], given its important role in the functioning of the immune system and antiviral defense. The idea of the present review is to highlight the role of zinc in strengthening immunity and to reveal the importance of medicinal plants via their content on bioactive molecules to prevent or to treat COVID-19.

## 2. Role of Zinc in the Immune System

Zinc is an essential trace element for humans, it is involved in many physiological functions, and its deficiency can impact human health. Zinc intake is guaranteed in humans, thanks to nutrition. The amount of zinc in the body of an adult is approximately 1.4–2.3 g, and it is the second most abundant ion after iron [12]. Zinc is essential for the activity of over 300 enzymes. It has multiple physiological roles. It is involved in the metabolism of proteins and fats [11].

**Figure 1:**
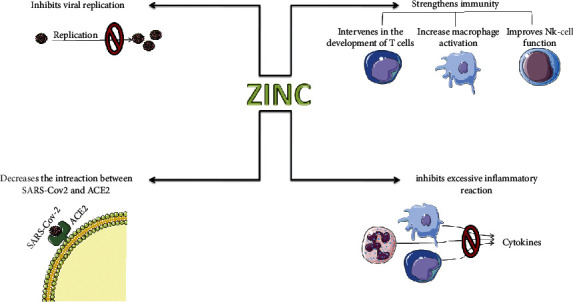
The role of zinc in the immune defense against SARS-CoV-2.

The immune system is influenced by zinc on various levels. On the one hand, zinc specifically alters immune functions, and on the other hand, the immune system which is a highly proliferative “organ” is influenced by zinc-dependent proteins involved in general cellular functions, i.e., replication, transcription, and signal transduction [13]. All cell subsets of the immune response are affected by zinc. Decreased zinc levels impair natural killer cell activity, phagocytosis by macrophages and neutrophils, and certain functions such as chemotaxis and the oxidative burst [14]. However, zinc is important even for the maturation and functioning of T cells, since zinc is an essential cofactor for the thymus hormone thymulin [15]. This hormone has intrathymic and peripheral immunoregulatory properties, and it is necessary for an intact thymus [16, 17]. Zinc deficiency thus leads to thymic atrophy; zinc also affects mature T cells. It induces the expression of the high-affinity receptor for interleukin-2, and zinc deficiency is associated with decreased T-cell proliferation after mitogen stimulation [18, 19]. Antibody production of B cells is also dependent on zinc. Interestingly, impaired antibody production can be restored through the addition of thymic cells, thus suggesting a T-cell-dependent defect [20]. Furthermore, the binding of zinc to immunoglobulins with as yet unclear functional relevance has been shown [21]. Additionally, it was reported that zinc can block the viral replication and prevent the excessive inflammatory reaction, and importantly, it was shown that zinc could react with the same receptor of SARS-CoV-2 (angiotensin-converting enzyme 2 receptor (ACE2)) and block its interaction with the spike proteins of the virus [22–24]. Overall, the clinical consequence of zinc deficiency is an impaired defense against bacterial, viral, and fungal infections (Figure 1).

## 3. Antiviral Activity of Zinc

The role of zinc in antiviral defense has been studied by several researchers through *in vitro* tests. Shishkov et al. have shown that zinc can specifically inactivate free varicella-zoster virus virions [25]. Zaslavsky revealed the inhibition of the viral RNA and protein synthesis of the vaccinia virus using zinc sulfate (ZnSO_4_) [26]. Similarly, Katz and Margalith showed that zinc chloride (ZnCl_2_) inhibits the RNA synthesis and viral yield of the vaccinia virus [27].

Likewise, Wei et al. have shown that zinc can inhibit the viral RNA and protein synthesis of transmissible gastroenteritis virus [28]. Additionally, it has been shown that zinc can be effective in inhibiting other viruses such as Sindbis virus, Semliki Forest virus, respiratory syncytial virus, human papillomavirus, human immunodeficiency virus, rhinovirus, herpes simplex virus, and hepatitis C virus. The mechanism of action of zinc against these viruses is variable as the inhibition of viral particle production and polyprotein cleavage, the inhibition of endosomal membrane fusion, the inhibition of viral polyprotein cleavage, the reduction in viral titer and plaque count, the stimulation of the proviral transcription factor activity, the inhibition of viral transcription and particle production, the inhibition of reverse transcriptase and viral protein synthesis, the inhibition of viral DNA polymerase, free virus inactivation, and the inhibition of RNA polymerase [23, 29–37]. Importantly, te Velthuis et al. have demonstrated that zinc can inhibit the replication of SARS-coronavirus (SARS-CoV) and equine arteritis virus (EAV) in cell culture [38].

The antiviral effect of zinc has been studied *in vivo* by many researchers via different routes of administration such as oral and topical. For instance, Godfrey et al. revealed that zinc reduced the severity of herpes simplex and the duration of treatment [39]. Similarly, Turner and Cetnarowski have shown that zinc reduced the duration of illness caused by rhinovirus [40]. In a randomized controlled trial, Murakami et al. studied the effect of zinc supplementation in chronic hepatitis C patients treated with pegylated interferon (PEG-IFN) alpha-2b plus ribavirin combination therapy; the comparison of the results obtained in the patients who received zinc and in the patients who did not receive it showed that the zinc supplementation decreased plasma thiobarbituric acid reactive substances, decreased the serum transaminases levels to within the normal range, and prevented the decrease of polyunsaturated fatty acids of erythrocyte membrane phospholipids [41]. Likewise, the results of a double-blind, placebo-controlled, clinical trial conducted by Eby et al. showed that zinc gluconate lozenges supplementation may reduce the symptom, the duration of treatment, and the severity of common cold [42].

Figure 1 was composed using Servier Medical Art templates, which are licensed under a Creative Commons Attribution 3.0 Unported License (http://smart.servier.com/).

## 4. The Effect of Zinc Supplementation in Respiratory Infections

Zinc is an essential micronutrient for the proper functioning of all organism cells; the body always requires a constant food supply of zinc because it does not store it. The problem of dietary zinc deficiency is very well known in developing countries [43]. The effect of zinc supplementation was studied as prophylactic or therapeutic strategies. Zinc supplementation in the therapeutic protocol of COVID-19 is supported by the results of many reports which have shown the beneficial effect of zinc supplementation against respiratory infections [44]. In a double-blind, randomized, controlled trial, the daily supplementation of 10 mg of elemental zinc for 609 children revealed a significant reduction of the morbidity of acute lower respiratory infection [45]. In a pilot study published by Abdulhamid et al., the results indicated that the oral intake of 30 mg/day of zinc reduced the number of days to treat children with oral antibiotics against cystic fibrosis [46]. Similarly, Rerksuppaphol and Rerksuppaphol conducted a randomized study in 64 hospitalized children with acute lower respiratory tract infections, and each child was randomly allocated to receive daily 30 mg of elemental zinc or placebo. The results showed that zinc supplementation reduced the duration of hospital stay and improved the recovery of children [47].

Several clinical trials involving zinc as either a preventative or combination therapy showed a positive recovery from COVID-19. For instance, a case report conducted by Finzi suggested that zinc salt lozenges administrated orally (maximum 200 mg) played a role in clinical recovery and showed a significant improvement of the COVID-19 symptoms [48]. Likewise, the results of a phase IIa double-blind, randomized controlled trial revealed the safety, the feasibility, and the ability of high-dose intravenous zinc treatment in rectifying the acute phase zinc deficiency seen in hospitalized COVID-19 patients [49]. In addition, it has been shown by Frontera et al. that zinc with an ionophore treatment is associated with a reduction in in-hospital mortality among adult COVID-19 patients [50].

## 5. Phytomedicines and COVID-19

### 5.1. Therapeutic Approaches against Virus Based on Medicinal Plants

Despite scientific advances, viral diseases remain a matter of concern. In addition to the vaccines that are established to immunize people, the search for bioactive molecules with antiviral properties does not cease to stop. Among the matrices most exploited in research, we find medicinal plants. The world flora is rich in a variety of medicinal plants used for its special properties against human illness. Many herbal remedies are rich in active ingredients which have a broad-spectrum antiviral activity [51]. In the past, the discovery of the antiviral activity of various herbal remedies was limited due to the highly infectious nature of viruses and the lack of suitable separation methods for screening the antiviral components of plants [52]. Several scientific publications have reported the antiviral activity of plants, such as the study conducted by Nolkemper et al. showed that aqueous extracts of plants species from the Lamiaceae family (*Rosmarinus officinalis*, *Melissa officinalis*, *Mentha x piperita*, *Prunella vulgaris*, *Salvia officinalis*, and *Thymus vulgaris*) exhibited antiviral effect against herpes simplex virus type 1 and type 2 and the most important against an acyclovir-resistant strain of HCV-1 (ACV^res^) [53]. The phytochemical analysis of these plants revealed their richness in polyphenolic compounds known by their antiviral activities such as rosmarinic acid, caffeic acid, apigenin, eriodictyol, and luteolin derivatives [54]. Likewise, Song et al. showed that the 3-galloyl group of catechin skeleton from the green tea (*Camellia sinensis*) plays an important role in the antiviral activity of this plant against influenza virus (A/H1N1, A/H3N2, and B virus) by the alteration of the physical properties of the viral membrane [55]. Additionally, Bayan et al. have shed light on the antiviral effect of the biologically active compounds of garlic (allicin, diallyl trisulfide, alliin, diallyl sulfide, and diallyl disulfide) against influenza A and B, HIV, HSV-1, viral pneumonia, cytomegalovirus, and rhinovirus [56].

Punicalagin is an active compound of pomegranate which had a virucidal property against influenza virus (influenza virus A; H3N2; H1N1 and influenza B); it inhibits the replication of the viral RNA. Interestingly, it has been shown that the combination of polyphenolic pomegranate extract and oseltamivir increases the anti-influenza effect of oseltamivir [57].

During the outbreak of COVID-19, various medicinal plant formulations recommended in traditional medicine have been used by people to alleviate symptoms associated with the SARS-CoV-2 infection. In a review study published by Luo et al., it was reported that the most used plants by 23 provinces in China for the prevention of COVID-19 were *Fructus forsythia*, *Radix astragali*, *Radix saposhnikoviae*, *Lonicerae japonicae flos*, *Rhizoma Atractylodis* macrocephalae, and *Radix glycyrrhizae* [58]. Likewise, Ulasli et al. have shown that the extracts of *Nigella sativa*, *Citrus sinensis*, and *Anthemis hyalina* can decrease the replication of SARS-CoV-2 and decrease the expression of the TRP genes family [59]. Similarly, in a study conducted by Lin et al., root water extract of *Isatis indigotica* and its five major compounds (indigo, indirubin, indican, sinigrin, and beta-sitosterol) were tested against 3C-like protease (3CLpro) of SARS-coronavirus, and the results demonstrated that the root extract and two major compounds (indigo and sinigrin) were efficient in the inhibition of 3CLpro of the virus [60] (Table 1).

### 5.2. Role of Plants' Secondary Metabolites

Secondary metabolites are groups of molecules that are essential for the adaptation of plants to their environment. They are divided into phenolics, terpenes, alkaloids, and steroids [61]. Among the most important secondary metabolites, which have a broad spectrum of biological effects, we found polyphenols, a family of organic molecules composed mainly of phenolic acids, and flavonoids, which are widely present in the plants [62], and they have many functional properties such as antioxidant activity [63], anticancer effect [64], and antiviral effect [65].

It was reported that phenolic compounds, such as quercetin, curcumin, and resveratrol, could modulate the expression of miRNAs in the host cells infected with SARS-CoV-2 [66]. Chiang et al. have demonstrated the antiviral effect of purified compounds from *Ocimum basilicum* (apigenin, linalool, and ursolic acid) against the following virus: herpes viruses (HSV), adenoviruses (ADV), hepatitis B virus, coxsackievirus B1 (CVB1), and enterovirus 71 (EV71) [67]. Similarly, it was found that carnosic acid isolated from the ethyl acetate fraction of *Rosmarinus officinalis* was effective against the human respiratory syncytial virus (hRSV) [68].

Caffeic acid and chlorogenic acid are two phenolic acid compounds found in *Plantago major*, the antiviral effect of this plant has been revealed against HSV-1, HSV-2, ADV-3, and ADV-11 viruses [69].

Baicalin is a flavonoid compound found in *Scutellaria baicalensis* Georgi [70]; it can inhibit the viral replication of human immunodeficiency virus type 1 (HIV-1) [71]. Recently, it was reported that this flavonoid exhibited potent antiviral activity against SARS-CoV-2 via the inhibition of SARS-CoV-2 3CL protease in a cell-based system [72].

Concerning the antiviral effect of terpenes compounds, it was reported that *Laurus nobilis* essential oil (main chemical constituents: *β*-ocimene, *α*-pinene, *β*-pinene, and 1,8-cineole) was effective against SARS-CoV (IC_50_ value = 120 ± 1.2 mg/ml) [73]. Sharifi-Rad et al. have demonstrated the antiviral effect of thymol, which is an aromatic compound from the monoterpene group, found in thyme essential oil, against herpes simplex virus type 1 (HSV-1) [74].

In a study published by Wen et al., ten diterpenoids, two sesquiterpenoids, and two triterpenoids were tested against SARS-CoV using a cell-based assay measuring SARS-CoV-induced cytopathogenic effect on Vero E6 cells. The results showed that these phytochemical constituents of essential oil have a significant anti-SARS-CoV effect [75].

Alkaloids are other types of secondary metabolites that contribute to the antiviral activity of plants. For instance, it was shown recently by Chen et al. the efficacy of lycorine, a toxic crystalline alkaloid found in plants belonging to the Amaryllidaceae family, against the Zika virus [76]. More importantly, Zhang et al. have proved the antiviral effect of lycorine and oxysophoridine (an alkaloid found in *Sophora alopecuroides*) against SARS-CoV-2 in cell culture by the inhibition of viral replication [77].

*Lycoris radiata* is another plant belonging to the Amaryllidaceae family, and it was reported that it is effective against SARS-CoV-2, thanks to its content on its main active compounds: lycorine and glycyrrhizin [78]. The last one is a triterpenoid saponin mainly found in *Glycyrrhiza glabra* roots; the antiviral effect of glycyrrhizin is well documented [79, 80]. Recently, it took the attention of many researchers to test it against SARS-CoV-2, and it was reported that it can bind to the same SARS-CoV-2 receptor (the angiotensin-converting enzyme 2 (ACE2) receptor) [80].

### 5.3. The Content of Medicinal Plants on Zinc

As previously signaled, zinc supplementation has a beneficial effect in strengthening the immune system; Table 2 provides a list of medicinal plants analyzed in various countries of the world for their zinc content. In Morocco, the following plants were analyzed: *Artemisia herba alba*, *Thymus vulgaris*, *Lavandula dentata*, *Rosmarinus tournefortii, Pistacia lentiscus*, *Pistacia lentiscus*, *Retama monosperma*, *Retama monosperma*, *Ziziphus spina-christi*, and *Ziziphus lotus*; the zinc content in these plants ranged between 28 ± 6 mg/kg in the pulp of *Ziziphus lotus* and 1016.73 mg/kg in leaves of *Rosmarinus tournefortii* [81–85]. Similarly, *Citrullus colocynthis*, *Balanites aegyptiaca*, *Solenostemma argel*, *Pergularia tomentosa*, *Acacia albida*, *Acacia ehrenbergiana*, and *Cymbopogon proximus* from Egypt were analyzed by Sheded et al. for the zinc content and revealed the following values: 25.87 ± 3.4 mg/kg, 15.47 ± 3 mg/kg, 73.77 ± 3.8 mg/kg, 58.47 ± 5.3 mg/kg, 30.27 ± 14.4 mg/kg, and 16.77 ± 4.4 mg/kg, respectively [86]. Ebrahim et al. found that the range of zinc content was between 3.34 ± 0.2 mg/kg and 58.80 ± 2.1 mg/kg in *Sesamum indicum*, *Olea europaea*, *Vaccinium myrtillus*, *Nigella sativa*, *Trigonella foenum-graecum*, *Pennisetum glaucum*, *Calligonum comosum*, *Citrullus colocynthis*, *Momordica charantia*, *Opuntia ficus-indica*, and *Haloxylon salicornicum* from Saudi Arabia [84]. Indian medicinal plants were studied by Pradhan et al., Datta et al., and Matev et al. [87–89]; the results showed a content in zinc ranged between 2.01 ± 0.1 mg/100 g in *Albizia lebbeck* seeds and 50 ± 1 mg/100 g in *Achyranthes aspera* (whole plant). Additionally, Pakistani plants were analyzed by Jabben et al. [90] and revealed a zinc content ranged between 22.33 ± 3.63 ppm in *Withania somnifera* and 65.85 ± 1.06 ppm in *Hordeum vulgare*. Plants from Turkey showed that the zinc content ranged between 53 mg/kg in *Punica granatum* fruits and 395.252 ± 12.92 mg/kg in *Ricinus communis* seeds [91, 92]. Similarly, 2.84 ± 0.005 mg/100 g of zinc was found in *Ficus capensis* from Nigeria [93]. The range of zinc in Romanian plants was between 20.83 ± 1.11 *μ*g/kg in *Lavandula angustifolia* and 64.76 ± 0.53 *μ*g/kg in *Althaea officinalis* [94], while the leaves of *Eruca sativa* from Italia and Bulgaria revealed a zinc content of 15.07 ± 0.60 mg/kg and 91.05 ± 0.65 mg/kg, respectively [95] (Table 2).

## 6. Conclusion

The world is facing a serious health crisis due to the emergence of a novel coronavirus (SARS-CoV-2). It is essential to find effective and safe solutions to reduce the morbidity and mortality caused by this pandemic situation. In this review, we discussed the evidence surrounding the role of zinc supplementation in strengthening immunity and in the recovery of patients with COVID-19. In addition, we proposed a list of medicinal plants as a source of bioactive molecules endowed with antiviral activities such as quercetin, curcumin, resveratrol, baicalin, allicin, punicalagin, lycorine, glycyrrhizin, and other secondary metabolites. This review will facilitate laboratory-based research and stimulate further analysis for the development of novel drugs to solve the current crisis.

## Figures and Tables

**Table 1:** Antiviral effect of medicinal plants.

Medicinal plant	Active compounds	Antiviral effect	Reference
*Rosmarinus officinalis*	Phenolic compounds	Herpes simplex virus type 1 and type 2 and acyclovir-resistant strain of HCV-1 (ACV^res^)	[53]
*Melissa officinalis*	Phenolic compounds	Herpes simplex virus type 1 and type 2 and acyclovir-resistant strain of HCV-1 (ACV^res^)	[53]
*Mentha x piperita*	Phenolic compounds	Herpes simplex virus type 1 and type 2 and acyclovir-resistant strain of HCV-1 (ACV^res^)	[53]
*Prunella vulgaris*	Phenolic compounds	Herpes simplex virus type 1 and type 2 and acyclovir-resistant strain of HCV-1 (ACV^res^)	[53]
*Salvia officinalis*	Phenolic compounds	Herpes simplex virus type 1 and type 2 and acyclovir-resistant strain of HCV-1 (ACV^res^)	[53]
*Thymus vulgaris*	Phenolic compounds	Herpes simplex virus type 1 and type 2 and acyclovir-resistant strain of HCV-1 (ACV^res^)	[53]
*Camellia sinensis*	Catechins ((−)-epigallocatechin gallate (EGCG), (−)-epicatechin gallate (ECG), and (−)-epigallocatechin (EGC))	A/H1N1, A/H3N2, and B virus	[55]
*Allium sativum*	Allicin, alliin, diallyl sulfide, diallyl disulfide	Influenza A and B, HIV, HSV-1, viral pneumonia, cytomegalovirus, and rhinovirus	[56]
*Punica granatum*	Punicalagin	Influenza virus A; H3N2; H1N1 and influenza B	[57]

**Table 2:** The zinc content of medicinal plants.

Name of plants	Country	Part used	Content in zinc	Reference
*Artemisia herba alba*	Morocco	Leaves	377.89 to 798.21 mg/kg	[81]
*Thymus vulgaris*	Morocco	Leaves + flowers	359.46 to 415.33 mg/kg	[81]
*Lavandula dentata*	Morocco	Leaves + flowers	480.39 mg/kg	[81]
*Rosmarinus tournefortii*	Morocco	Leaves	1016.73 mg/kg	[81]
*Justicia adhatoda* L.	Pakistan	Whole plant	31.64 ± 7.84 ppm	[90]
*Achyranthes aspera* L.	Pakistan	Whole plant	20.91 ± 4.61 ppm	[90]
*Alternanthera pungens* Kunth	Pakistan	Whole plant	37.86 ± 2.76 ppm	[90]
*Parthenium hysterophorus* L.	Pakistan	Whole plant	28.92 ± 9.18 ppm	[90]
*Cannabis sativa* L.	Pakistan	Leaves	29.45 ± 4.81 ppm	[90]
*Ricinus communis* L.	Pakistan	Whole plant	31.55 ± 4.20 ppm	[90]
*Hordeum vulgare* L.	Pakistan	Seeds	65.85 ± 1.06 ppm	[90]
*Withania somnifera* (L.) Dunal	Pakistan	Whole plant	22.33 ± 3.63 ppm	[90]
*Citrullus colocynthis*	Egypt	Whole plant	25.87 ± 3.4 mg/kg	[86]
*Balanites aegyptiaca*	Egypt	Whole plant	15.47 ± 3.0 mg/kg	[86]
*Solenostemma argel*	Egypt	Whole plant	73.77 ± 3.8 mg/kg	[86]
*Pergularia tomentosa*	Egypt	Whole plant	58.47 ± 5.3 mg/kg	[86]
*Acacia albida*	Egypt	Whole plant	30.27 ± 14.4 mg/kg	[86]
*Acacia ehrenbergiana*	Egypt	Whole plant	16.77 ± 4.4 mg/kg	[86]
*Cymbopogon proximus*	Egypt	Whole plant	25.77 ± 5.3 mg/kg	[86]
*Sesamum indicum*	Saudi Arabia	Seeds	54.90 ± 1.9 mg/kg	[84]
*Olea europaea*	Saudi Arabia	Leaves	7.79 ± 0.5 mg/kg	[84]
*Vaccinium myrtillus*	Saudi Arabia	Fruit	3.34 ± 0.2 mg/kg	[84]
*Nigella sativa*	Saudi Arabia	Seeds	58.80 ± 2.1 mg/kg	[84]
*Trigonella foenum-graecum*	Saudi Arabia	Seeds	29.80 ± 1.3 mg/kg	[84]
*Pennisetum glaucum*	Saudi Arabia	Seeds	34.00 ± 1.5 mg/kg	[84]
*Calligonum comosum*	Saudi Arabia	Whole plant	11.80 ± 0.6 mg/kg	[84]
*Citrullus colocynthis*	Saudi Arabia	Fruit	12.80 ± 0.6 mg/kg	[84]
*Momordica charantia*	Saudi Arabia	Fruit	21.20 ± 1.1 mg/kg	[84]
*Opuntia ficus-indica*	Saudi Arabia	Fruit	14.30 ± 0.7 mg/kg	[84]
*Haloxylon salicornicum*	Saudi Arabia	Whole plant	6.67 ± 0.4 mg/kg	[84]
*Pistacia lentiscus*	Morocco	Leaves	230.36 mg/kg	[82]
*Pistacia lentiscus*	Morocco	Fruits	168.11 mg/kg	[82]
*Retama monosperma*	Morocco	Branches/leaves	140.91 mg/kg	[83]
*Retama monosperma*	Morocco	Seeds	44.33 mg/kg	[83]
*Ziziphus spina-christi*	Morocco	Fruit	0.44 ± 0.06 mg/100 g	[85]
*Ziziphus spina-christi*	Morocco	Pulp	0.38 ± 0.03 mg/100 g	[85]
*Ziziphus spina-christi*	Morocco	Seed	0.65 ± 0.04 mg/100 g	[85]
*Ziziphus spina-christi*	Morocco	Almond	0.86 ± 0.05 mg/100 g	[85]
*Ziziphus lotus*	Morocco	Fruit	0. 31 ± 0.01 mg/100 g	[85]
*Ziziphus lotus*	Morocco	Pulp	0.28 ± 0.06 mg/100 g	[85]
*Ziziphus lotus*	Morocco	Seed	0.59 ± 0.04 mg/100 g	[85]
*Ziziphus lotus*	Morocco	Almond	0.78 ± 0.04 mg/100 g	[85]
*Amaranthus viridis*	India	Leaves	9.73 ± 1.02 mg/100 g	[87]
*Chenopodium album*	India	Leaves	8.44 ± 0.9 mg/100 g	[87]
*Diplazium esculentum*	India	Leaves	2.73 ± 0.1 mg/100 g	[87]
*Nasturtium officinale*	India	Leaves	2.04 ± 0.03 mg/100 g	[87]
*Urtica dioica*	India	Leaves	2.32 ± 0.04 mg/100 g	[87]
*Allium neapolitanum*	Turkey	Bulb	234.969 ± 9.04 mg/kg	[91]
*Allium scorodoprasum*	Turkey	Bulb	386.150 ± 12.84 mg/kg	[91]
*Cichorium intybus*	Turkey	Root	257.858 ± 5.48 mg/kg	[91]
*Ferula communis*	Turkey	Rhizome	302.083 ± 6.18 mg/kg	[91]
*Glycyrrhiza glabra*	Turkey	Rhizome	182.978 ± 2.93 mg/kg	[91]
*Laurus nobilis*	Turkey	Seed	281.449 ± 5.86 mg/kg	[91]
*Paliurus spina-christi*	Turkey	Fruit	177.784 ± 6.91 mg/kg	[91]
*Papaver somniferum*	Turkey	Seed	330.341 ± 4.09 mg/kg	[91]
*Pinus brutia*	Turkey	Resin	174.993 ± 5.83 mg/kg	[91]
*Pistacia terebinthus*	Turkey	Fruits	173.171 ± 6.86 mg/kg	[91]
*Quercus infectoria*	Turkey	Gall	166.910 ± 2.69 mg/kg	[91]
*Rhus coriaria*	Turkey	Seed	307.730 ± 6.94 mg/kg	[91]
*Ricinus communis*	Turkey	Seed	395.252 ± 12.92 mg/kg	[91]

## Data Availability

The data used to support the findings of this study are included within the article.
